# Assessment of access and outcomes of kidney transplantation through the reforms of the Swiss organ allocation system

**DOI:** 10.3389/fpubh.2024.1500781

**Published:** 2025-01-08

**Authors:** Massimiliano Bertacchi, Sylvie Ferrari-Lacraz, Jakob Nilsson, Agim Thaqi, Yvan Schmutz, Caroline Wehmeier, Thomas Schachtner, Thomas Mueller, Dela Golshayan, Julien Vionnet, Stefan Schaub, Fadi Haidar, Isabelle Binet, Jutta Thierbach, Urs Wirthmueller, Daniel Sidler, Franz Immer, Jean Villard

**Affiliations:** ^1^Nephrology and Hypertension Division, Geneva University Hospitals, Geneva, Switzerland; ^2^Transplant Immunology Unit, Geneva University Hospitals, Geneva, Switzerland; ^3^Department of Immunology, University Hospital Zurich, Zurich, Switzerland; ^4^Federal Office of Public Health, Bern, Switzerland; ^5^Analytica SA, Lausanne, Switzerland; ^6^Nephrology and Transplant Immunology Division, University Hospital Basel, Basel, Switzerland; ^7^Nephrology Division, University Hospital Zurich, Zurich, Switzerland; ^8^Transplantation Center, Centre Hospitaliser Universitaire Vaudois, Lausanne, Switzerland; ^9^Division of Immunology and Allergy, Centre Hospitaliser Universitaire Vaudois, Lausanne, Switzerland; ^10^Clinic for Nephrology and Transplantation Medicine, Kantonsspital St. Gallen, St. Gallen, Switzerland; ^11^Blood Transfusion Service Eastern Switzerland, Swiss Red Cross, St. Gallen, Switzerland; ^12^Department of Laboratory Medicine, Inselspital, Bern University Hospital, Bern, Switzerland; ^13^Department of Nephrology and Hypertension, Inselspital, Bern University Hospital, Bern, Switzerland; ^14^Swisstransplant, The Swiss National Foundation for Organ Donation and Transplantation, Bern, Switzerland

**Keywords:** kidney transplantation, allocation system, transplant immunology, allocation fairness, hyperimmunized patients, waiting time

## Abstract

**Introduction:**

The Swiss allocation system for kidney transplantation has evolved over time to balance medical urgency, immunological compatibility, and waiting time. Since the introduction of the transplantation law in 2007, which imposed organ allocation on a national level, the algorithm has been optimized. Initially based on waiting time, HLA compatibility, and crossmatch performed by cell complement-dependent cytotoxicity techniques, the system moved in 2012 to a score including HLA compatibility, waiting time, anti-HLA antibodies detected by the Luminex^®^ technology, and a virtual crossmatch. In 2015, the score was optimized to balance the impact of preemptive listing and HLA matching of hyperimmunized recipients.

**Methods:**

We reviewed access to transplants and post-transplant outcomes along those changes, defining three periods (v0: 2007–2012, v1: 2012–2015, v2: 2015–2020).

**Results:**

Changes in the Swiss allocation system improved the fairness of access to transplantation, particularly for hyperimmunized patients. However, the system still fails to grant fair access to some blood groups. Furthermore, our data showed that rule modifications did not impact early post-transplant complications, maintaining similar time to first rejection and 1-year graft survival across subgroups.

**Discussion:**

Such an analysis is useful for validating changes made to the allocation system and identifying aspects that need to be implemented in future revisions.

## Introduction

Transplantation remains the optimal treatment for patients suffering from end-stage kidney (ESKD) disease, with lower mortality and morbidity compared to kidney replacement therapy (1, 2). However, poor organ availability, geographical limitations, and the presence of hyperimmunized patients represent a worldwide challenge to a fair distribution of available organs (3). In this setting, effective allocation systems are essential and several approaches, with multiple parameters used to define allocation priorities, have been developed in different countries (4, 5).

In 2022, 570 patients received solid organ transplantation in Switzerland, 342 of which were kidney transplants. However, at the end of the same year, 2,150 patients were still waiting for an organ, 1,435 of whom for a kidney.^1^

Based on the revision of the Swiss federal law on solid organ transplantation in 2007, the Swiss national program for solid organ transplantation replaced the existing regional allocation system. Like other allocation systems, such as the American KAS (6, 7), ETKAS for the Eurotransplant consortium (8), or the French KAS (9), the Swiss allocation system aimed to find a balance between a utilitarian allocation system and fair transplant access for recipients on the waiting list, reducing geographic disparities, prioritizing recipients who need urgent transplantation, minimizing HLA mismatch in young recipients (priority for recipients <20y-o.), and grant an equal distribution of available organs from deceased donors.

**Figure 1:**
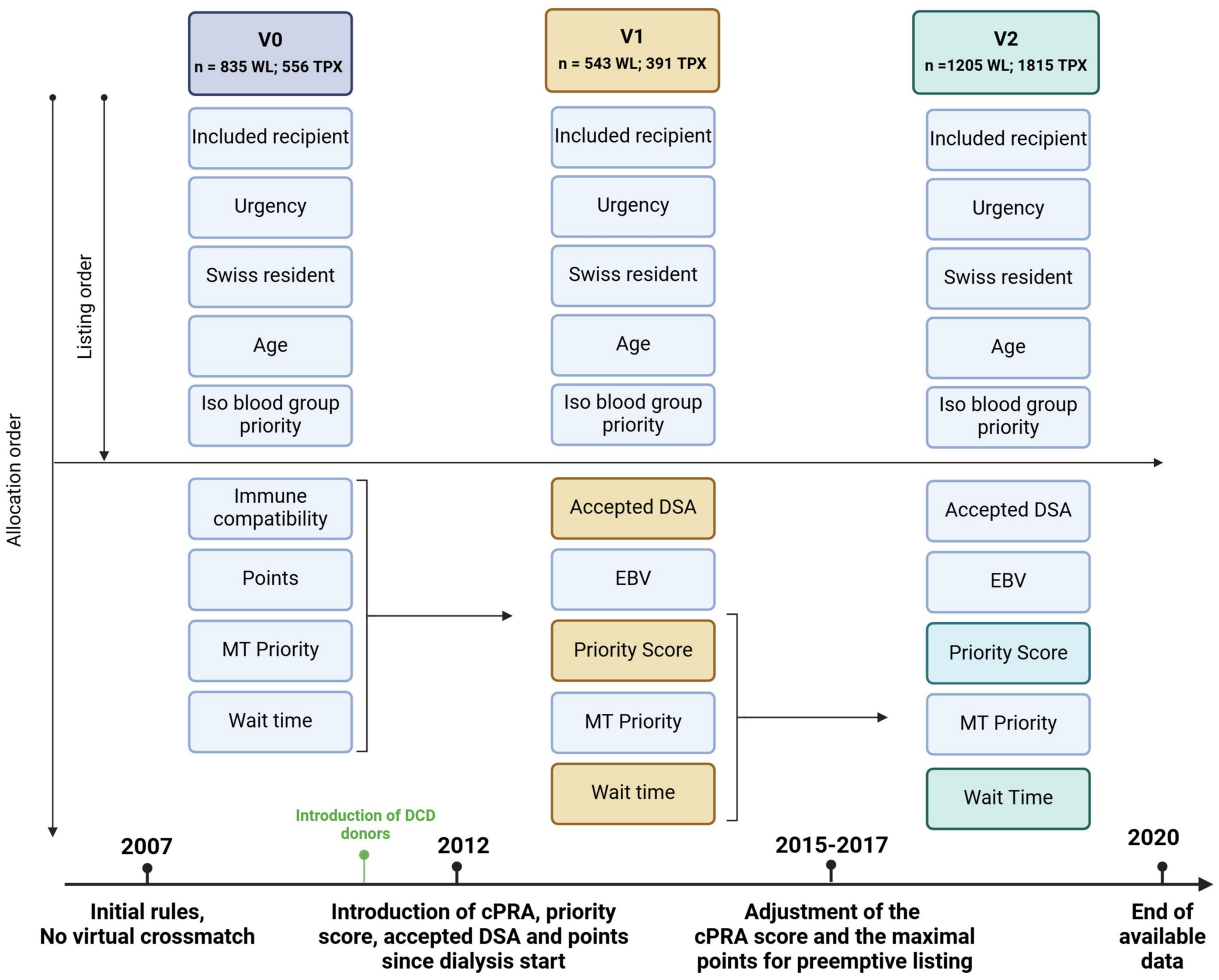
Evolution of the Swiss kidney allocation rules since the introduction of the Swiss national allocation system in 2007 (the last period includes the last two law revisions). The allocation system is based on sequential inclusion of priority criteria; if no compatible patient fulfills the first criteria or more than one patient fulfills the criteria (for example two urgent transplants), the second criterion applies to discriminate between them and so on. WL, waiting list; TPX, Transplanted patients; DCD, Organ donation after cardiocirculatory death Accepted DSA = DSA accepted for patients with a cPRA >98. EBV priority = priority is given for EBV – recipients if EBV – donor. Priority score = point system considering waiting time, HLA matching and cPRA MT priority = multiple organ transplantation are prioritized over single organ transplantation.

The Swiss allocation system was initially based on an algorithm balancing emergency criteria, age, waiting time, priority for multi-organ transplantation, and expected medical benefit based on blood group and immune compatibility (Figure 1). Compared to the American allocation system (10), the Swiss algorithm does not consider kidney quality (other than donor age and immune compatibility) in the allocation process.

Since 2007, similarly to the experience of other countries (9), the rules regulating kidney allocation have been modified several times. On the one hand, following the technical advances in detecting recipients’ anti-HLA antibodies and, on the other hand, optimizing allocation fairness, with prioritization of hyperimmunized patients, aiming toward an optimal balance between a reasonable rate of organ proposal and sufficient tissular compatibility and transplantation outcome. The history of changes to the Swiss Allocation Act is available at www.fedlex.admin.ch (Act: 810.212.41) and are summarized in Supplementary Table S1.

Before 2012, the patient’s immunological status was evaluated via a panel reactive antibody (PRA) test in order to estimate the percentage of potential compatible donors, which was based on a complement-dependent cytotoxicity (CDC) assay with standardized plates (11, 12).

Since 2011 organ donation after cardiocirculatory death DCD donors was implemented in Switzerland with a progressive increase in the number of DCD donors since 2012.

In 2012, the introduction of Luminex (13) allowed the direct assessment of patients’ anti-HLA antibodies without the need for an indirect deduction via the cytotoxicity assay. The use of Luminex^®^ permitted the introduction of a virtual crossmatch and the use of a calculated PRA (cPRA) (14), representing the theoretical percentage of compatible donors based on the database of donors of the Swiss allocation system. The possibility of quantifying anti-HLA antibodies opened the possibility of accepting organs across DSA. As graft survival was shown to be inversely correlated to peak PRA-HLA strength (15), with DSA-MFI higher than 900 strongly predictive for antibody-mediated rejection (16), the avoidance of DSA became essential, and the allocation of well-matched organs to hyperimmunized patients a priority. For this reason, an MFI cut-off of 1,000 was defined as relevant, and the concept of *accepted DSA* was introduced for hyperimmunized patients with a PRA >95%.

Following the development of the Luminex^®^ technology in every HLA laboratory in Switzerland, the National Reference Laboratory for Histocompatibility (LNRH at HUG) organizes quality controls for these laboratories every year, leading to the standardization of the technology. The new allocation system was, therefore, based on the specification of the risk according to the cPRA and the virtual crossmatch: allocation around DSA (for HLA-A, -B, and -DR loci) and the equitable allocation of kidneys among the different priority groups (according to the cPRA level). A priority score was developed based on the waiting time (years since the start of dialysis), the HLA matching score (on loci -DR, −A and -B), and the cPRA score (Supplementary Table S2). The initial priority score was modulated on simulations estimating the distribution of offers to recipients with different cPRA (Supplementary Figure S1). The priority score was adapted in the following years. After an evaluation in 2013 and 2014, factors of the cPRA score were corrected in 2015, improving the redistribution of kidney allocation. In 2017, a maximum waiting time for patients preemptively listed before the need for dialysis was introduced to correct for a potential advantage of hyperimmunized patients.

All these changes aimed to improve the equal distribution of organs for recipients on the waiting list. Nevertheless, those changes might have impacted some subpopulations regarding access to transplant and post-transplant outcomes. In particular, transplantation across DSA or lower HLA matching in highly sensitized patients might have affected transplantation outcomes (16).

The effect of the introduction of the Swiss national allocation system over the regional one on heart (17), lungs (18), and liver (19) has been studied in the past, but no study analyzed the impact of rule changes in kidney allocation. In this study, we report changes in access to transplantation (considering the time to transplantation) and early transplant complications (considering episodes of first rejection and the 1-year graft survival) across progressive low changes in the Swiss kidney allocation system.

## Materials and methods

In this study, we performed a retrospective analysis, including patients on the waiting list from all Swiss transplantation centers for a kidney transplantation between January 1st, 2007, and June 30st, 2020. Patients listed for combined organ transplantation and patients who received a kidney from a living donor were excluded from the analysis.

Data were available from two databases: the Swiss Transplant Cohort Study (STCS) (20), with data on recipients’ characteristics and the Swiss National Organ Allocation System (SOAS) database, with data on patients removed from the waiting list without transplantation or patients still on the waiting list on June 30st, 2020. Anonymized data of the two datasets were matched by soascaseID and coded. A list of data available on the two datasets is available in Supplementary Table S3. Additionally, we analyzed some donor populational data available through the annual reports of Swisstranplant.^2^

We assessed access to transplants and post-transplant outcomes along three periods (v0: 2007–2012, v1: 2012–2015, v2: 2015–2020) defined by the changes in the allocation rules described above (Figure 1). Because of the minor changes in the last revision and the shortness of the last two periods, changes in 2015 and 2017 were considered in the same periods (v2). Recipients were assigned to one of the three periods depending on the time they were removed from the waiting list, with or without transplantation, according to the applied allocation rule at that time.

We identified six variables possibly impacting the waiting time for transplantation: age (adults vs. children defined by age ≤ 20 years old), ABO blood groups, dialysis modality (Hemodialysis (HD) vs. peritoneal dialysis (PD) vs. patients preemptively listed), causes of ESKD (classified in 9 different diagnoses: congenital and hereditary diseases; diabetic nephropathy; glomerulonephritis; interstitial nephritis; hypertensive nephroangiosclerosis; polycystic disease; pyelonephritis or vesicoureteral reflux, previous graft failure, and other unspecified diagnosis), level of immunization (low immunized vs. highly-immunized recipients defined by a peak PRA ≥85% vs. very highly immunized recipients defined by a PRA ≥ 98%) (21), and transplantation across DSA (for the HLA loci -A, -B, -C, -DR, -DQ, -DP).

To evaluate access to transplantation and transplantation outcome in the three periods, patients were divided into subgroups defined by those six variables.

We described the changes in donors and recipients characteristics over the three periods, assessing the transplantation rate between different groups over the three periods. We then performed a time-to-event analysis considering the total waiting time and time to first rejection in each period looking for disadvantaged recipients.

The influence of rule changes across periods was estimated by an eventual inhomogeneity in Pearson’s chi-squared test or indirectly assumed by the persistence or vanishing of disadvantaged recipients (identified by longer waiting times or shorter time to rejection in the time-to-event analysis) along the three periods.

Because of the limited data on non-transplanted patients only the time to occurred transplantations could be analyzed. Because of the different patients’ characteristics and time lengths of the three periods, the time-to-event analysis was performed only within each period and not across them. A time-to-event analysis in each group and across periods was performed for the time to rejection (censured for rection in the first year post-transplantation). Finally, we analyzed the 1-year graft survival in each period.

### Statistics

Descriptive statistics for donors and recipients were reported as the total number and percentage of patients for each subgroup and period. Continuous variables were reported as mean and standard deviation. A Pearson’s chi-squared test was used to analyze the rate of transplant recipients considering patients’ characteristics across periods.

The study’s variables were analyzed using a Q-Q plot, and their distribution was tested for normality using a Shapiro–Wilk test. None of our variables were normally distributed, confirming the necessity of non-parametric tests for the statistical analysis.

Donor’s characteristics and the mean total time to transplantation through the three periods were analyzed with a variance analysis (ANOVA on ranks).

To evaluate access to transplantation, we performed a left-censured time-to-event analysis considering the total time on the waiting list until transplantation, using a log-rank test and a modified Kaplan–Meier plot.

Because of the high number of correlations in the time-to-event analysis (with a 3×6 table), significant *p*-values were tested with a Bonferroni correction (Supplementary Table S4). Unless specified with an *, the uncorrected p-values were reported in the text.

We performed a multivariate hazard model to estimate the impact of each independent variable on time to transplantation. The impact of peak-PRA (independent variable) on time to transplantation (dependent variable) was assessed with a linear regression.

The outcome of accomplished transplantations was analyzed by considering the time to first rejection in the first year, performing a time-to-event analysis (log-rank test) and a cox-regression, and comparing the 1-year graft survival through a Pearson’s chi-squared test.

## Results

Our cohort consists of 2,583 patients on the waiting list for a single kidney transplantation between 01.07.2007 and 31.12.2019 who were either successfully transplanted (*n* = 1815) or removed from the list (*n* = 768), either because of death (*n* = 300) or because of a definitive transplant contraindication (Table 1A). A total of 1,469 patients were still on the waiting list at the end of v2 and were not considered in our cohort (left-censored). Fifteen patients were excluded from the analysis because of incomplete data. The number of donors per year increased in v2 compared to v0 and v1 (118/y in v0, 95/y in v1, 143/y in v2), as well as the number of transplant patients per year (113/y in v0, in 130/y v1, 156/y in v2).

**Table 1:** Cohort demographics of (A) all patients on the waiting list from 01.07.2007 to 31.12.2019, (B) donors, and (C) patients successfully transplanted in the different historical periods (defined by the time of list removal). (D) Mean time to transplantation (with total and active waiting time), time to rejection, and overall follow-up. (E) Rate of early graft complications in the 1st year post-transplantation.

		Historical periods (based on transplantation date)				
		Total	v0	v1	v2	*p*-value
(A) Patients on the waiting list—Mean n/y (total n)		185.5 (2583)	167.0 (835)	233.7 (543)	200.8 (1205)	
Reason for list removal - n (%)	Transplanted	1815 (70%)	566 (68%)	391 (72%)	858 (71%)	0.153**
	Removed w/o transplantation	768 (30%)	269 (32%)	152 (28%)	347 (29%)	
Death on List—%		4.0	7.8	3.5	1.7	**<0.0001**
(B) Donors—Mean n/y (total n)		118.0 (1661)	94.8 (474)	107.7 (327)	143.3 (860)	
Donor type - n (%)						
DBD		1,391 (84%)	471 (99.4%)	294 (90%)	626 (73%)	<0.0001**
DCD		270 (16%)	3 (0.6%)	33 (10%)	234 (27%)	
Sex—Male %		52.8	56.7	57.4	47.8	0.207*
Age—Mean (y)	53.9	52.0	52.3	55.8	0.037*	
(C) Transplanted patients—Mean n/y (total n) * v2 over 5.5 years		134.4 (1815)	113.2 (566)	130.3 (391)	156.0 (858)*	
Age—Mean ± SD		53.15 ± 14.9	52.42 ± 14.5	52.3 ± 15.5	54.02 ± 14.8	0.033*
Sex—n (%)	Female	687 (38%)	188 (33%)	158 (40%)	341 (40%)	0.023**
	Male	1,128 (62%)	378 (67%)	233 (60%)	517 (60%)	
Age group—n (%)	Adult	1724 (95%)	536 (95%)	367 (94%)	821 (96%)	0.369**
	Child	91 (5%)	30 (5%)	24 (6%)	37 (4%)	
Dialysis type—n (%)	None	94 (5%)	34 (6%)	18 (5%)	42 (5%)	0.007**
	HD	1,423 (79%)	445 (79%)	328 (84%)	650 (76%)	
	PD	298 (16%)	87 (15%)	45 (11%)	166 (19%)	
ABO group—n (%)	A	1,155 (45%)	383 (46%)	209 (39%)	563 (47%)	0.029**
	AB	107 (4%)	30 (4%)	19 (3%)	58 (5%)	
	B	293 (11%)	105 (12%)	58 (11%)	130 (11%)	
	O	1,028 (40%)	317 (38%)	257 (47%)	454 (37%)	
Transplant across DSA —n (%)	Yes	105 (6%)	-	55 (14%)	50 (6%)	0.018**
	No	1710 (94%)	-	336 (86%)	808 (94%)	
(D) Mean time—days ±SD						
To transplantation (total)		1024.6 ± 641	907.5 ± 687	1148.7 ± 685	1045.2 ± 642	<0.0001*
To transplantation (active)		649.3 ± 678	684.4 ± 735	724.0 ± 742	591.4 ± 598	<0.0001*
To rejection		324.1 ± 565	399.5 ± 715	360.5 ± 582	224.5 ± 300	0.677*
Follow-up		507.8 ± 804	795.9 ± 1,074	554.9 ± 759	240.0 ± 350	<0.0001*
(E) Early post-transplant complications						
1-Y-rejection-free rate n° (%)		1,412 (80%)	401 (72)%	301 (80%)	689 (86%)	<0.0001**
1-Y graft survival n° (%)		1751 (97%)	542 (96%)	379 (97%)	830 (97%)	0.053

Continuous values, as age and time to transplantation were compared with a one-way ANOVA on ranks (*). The repartition of patients in the different sub-groups were compared with a Pearson’s chi-squared test (**). DBD, donation after brain death; DCD, donation after cardiocirculatory death; HD, Hemodialysis; PD, Peritoneal dialysis. Significant *p*-values are highlighted in bold.

Donors’ characteristics changed across periods (Table 1B), with older donors in v2 (+3.5 years; *p* = 0.037) and with an increase in the number of donors and CDC donors in v1 and v2 (*p* < 0.001). The rate of donors with blood group A vs. other blood groups and the sex distribution remained similar in the three periods (*p* = 0.207 and *p* = 0.177, respectively).

Transplanted recipients (Table 1C) were mainly males (62%), with a mean age of 53. 95% were adults (*n* = 1735), and 6.3% (*n* = 132) were considered hyperimmunized recipients (either highly- or very highly immunized; *n* = 87 and *n* = 45).

On average, patients spent 1,025 ± 641 total- and 649 ± 678 active days on the waiting list before transplantation. The mean time-to-transplantation differed in the three periods for total and active time (*p* < 0.0001 and *p* < 0.0001 respectively).

The rate of transplanted patients over patients removed from the list without transplantation slightly improved in v1 (72%) and v2 (71%) compared to v0 (68%) but without reaching statistical significance (Pearson’s *p* = 0.153; Table 1A). We observed a significant reduction in the death rate on list along the three periods (7% in v0, 3.5% in v1, 1.7% in v2; *p* < 0.0001).

The mean follow-up of transplanted recipients was 508 ± 804 days and was significantly longer in v0 compared to v1 and v2 (respectively 795.9 ± 1,074; 554.9 ± 759; 240.0 ± 350 days, *p* < 0.0001).

### Access to transplantation

The distribution of transplanted recipients across periods remained stable between children and adults (Chi-Square 0.369). We observed an increase in transplanted female to male rate in v1 and v2 compared to v0 (40% vs. 33%). The rate of preemptive transplantation remained similar in the three periods, but we observed a small variation in the distribution between HD and PD across periods (HD 79% in v0, 84% in v1, 76% in v2), but with HD remaining the preferred modality. Concerning the blood groups, we observed a prevalence of Group A recipients in the three periods, with a transient increase in Group 0 recipients in v1.

**Figure 2:**
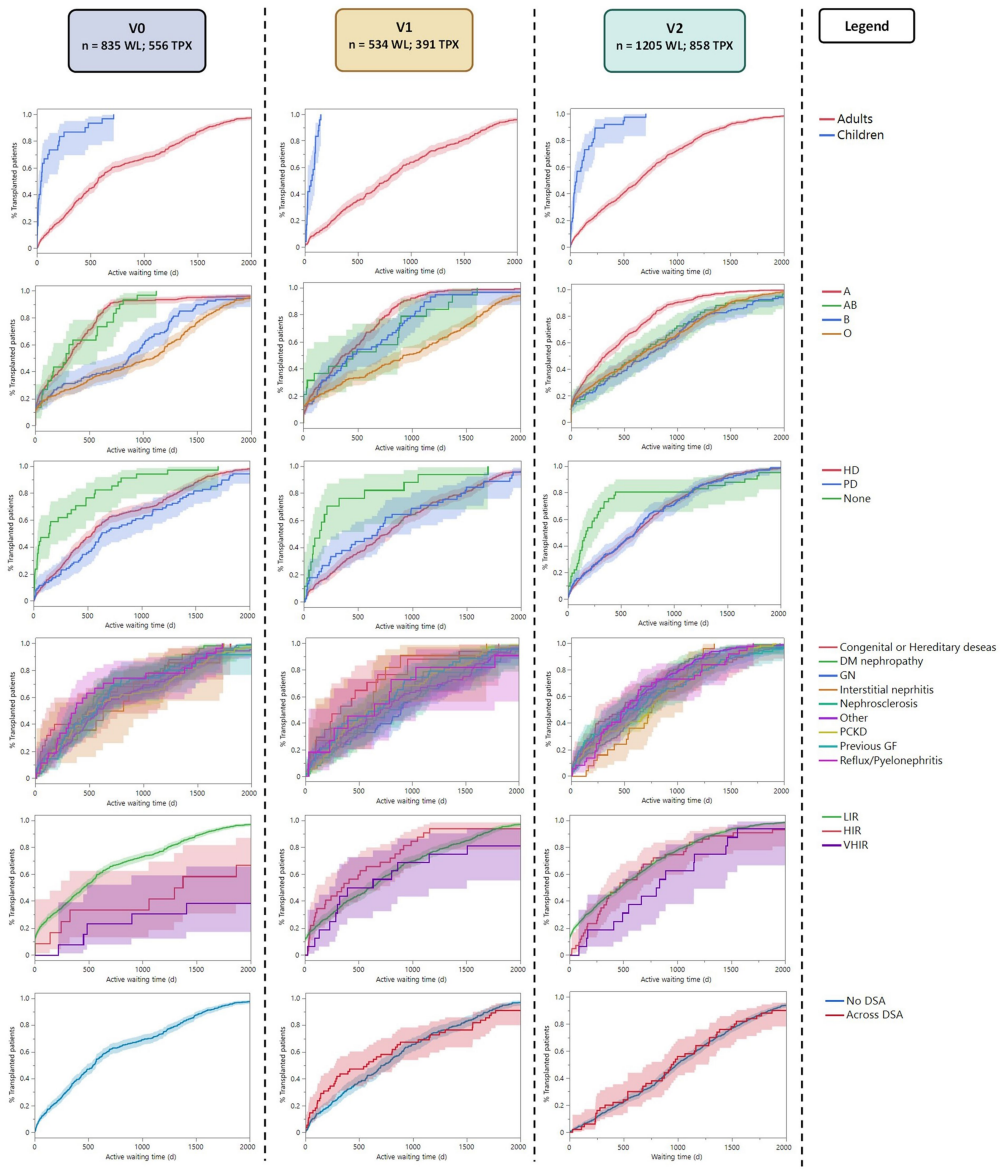
Reverse Kaplan–Meier plot showing the active time to transplant analysis for each subgroup along the three periods. LIR, Low Immunized Recipients (PRA <85); HIR, High Immunized Recipients (PRA ≤85 and < 98); VHIR, Very High Immunized Recipients (PRA ≥98).

Considering the active time to transplantation (Table 2A; Figure 2), we observed that children were transplanted 7.4 times faster compared to adults in all three periods (*p* < 0.0001). Considering the recipient’s blood groups, a significant disparity in time to transplantation was observed in all three periods (*p* < 0.0001), with Group A recipients transplanted 4.5, 3.9, and 2.2 times faster than Group 0 recipients in the three periods (*p* < 0.0001). The overall impact of other blood groups varied in the three periods (Table 2A; Supplementary Table S5).

**Table 2:** Time-to-event analysis considering (A) time to transplantation in different sub-groups along the three periods (Log Rank test), (B) time to rejection (Log Rank test), and (C) 1Y graft survival analysis (Pearson’s Chi Square Test).

		Historical periods (p-values)		
		v0	v1	v2
(A) Active time to transplant analysis				
	Age group	**< 0.0001**	**< 0.0001**	**< 0.0001**
	ABO blood group	**< 0.0001**	**< 0.0001**	**< 0.0001**
	ESKD diagnosis	0.685	0.138	**0.029**
	Dialysis modality	**< 0.0001**	**<0.001**	0.460
	Immunity group	**< 0.0001**	0.112	0.156
	Transplantation across DSA	–	0.320	0.345
(B) Time to rejection				
	Age group	0.172	0.285	0.160
	ABO blood group	0.142	0.601	0.341
	Dialysis Modality	0.580	0.340	0.525
	ESKD diagnosis	0.401	0.757	0.089
	Immunity group	0.107	0.019*	0.648
	Transplantation across DSA	–	0.880	0.130
(C) 1Y graft survival				
	Age group	0.800	0.748	0.253
	ABO blood group	0.110	0.611	0.731
	ESKD diagnosis	0.125	0.826	0.793
	Dialysis modality	0.549	0.681	0.211
	Immunity group	0.477	0.173	0.481
	Transplantation across DSA	-	0.792	0.764

*Not significant after Bonferroni correction. Significant *p*-values are highlighted in bold.

Recipients preemptively transplanted without kidney replacement therapy were transplanted 1.7 times faster than recipients on HD and 2.6 times faster than recipients on PD in v0 (*p* < 0.0001). This advantage seems to persist in v1 (*p* < 0.001) even if the hazard ratios between preemptive transplantation and dialysis (HD or PD) did not show any significant difference in this period (Supplementary Table S5). The advantage disappeared in v2 (*p* = 0.460). No difference was observed between recipients on HD vs. PD before transplantation. Those results remained significant after the Bonferroni correction (Supplementary Table S4).

Low-immunized recipients (with a peak PRA <85%) were transplanted six times faster compared to hyperimmunized recipients in v0 (*p* < 0.0001), in particular, 4 times faster than highly immunized patients (*p* < 0.001) and 10 times faster than very highly immunized patients (*p* < 0.0001). This advantage disappeared in v1 and v2. The linear regression between peak-PRA and time to transplantation showed no significant correlation in the three periods (*p* = 0.424, R2 = 0.001; *p* = 0.005, R2 = 0.02; *p* = 0.285, R2 = 0.001; respectively).

Considering the diagnosis leading to kidney failure, there was no difference in time to transplantation in v0 and v2 (*p* = 0.107, *p* = 0.648, respectively). In v1, the Pearson Chi-square test showed a possible difference, with slightly faster transplantation for congenital diseases, but this was not significant after the Bonferroni correction (*p* = 0.342*). Similarly, the presence of preformed DSA at transplantation (available only in v1 and v2) did not influence the time to transplantation in both periods (*p* = 0.320 and *p* = 00.345).

### Clinical outcomes after transplantation

The mean time to rejection was 324.1 ± 565 days. The overall 1-year graft survival was of 97%. Both time to rejection and graft survival were similar in the three periods (Pearson’s chi-squared *p* = 0.677 and *p* = 0.053).

**Figure 3:**
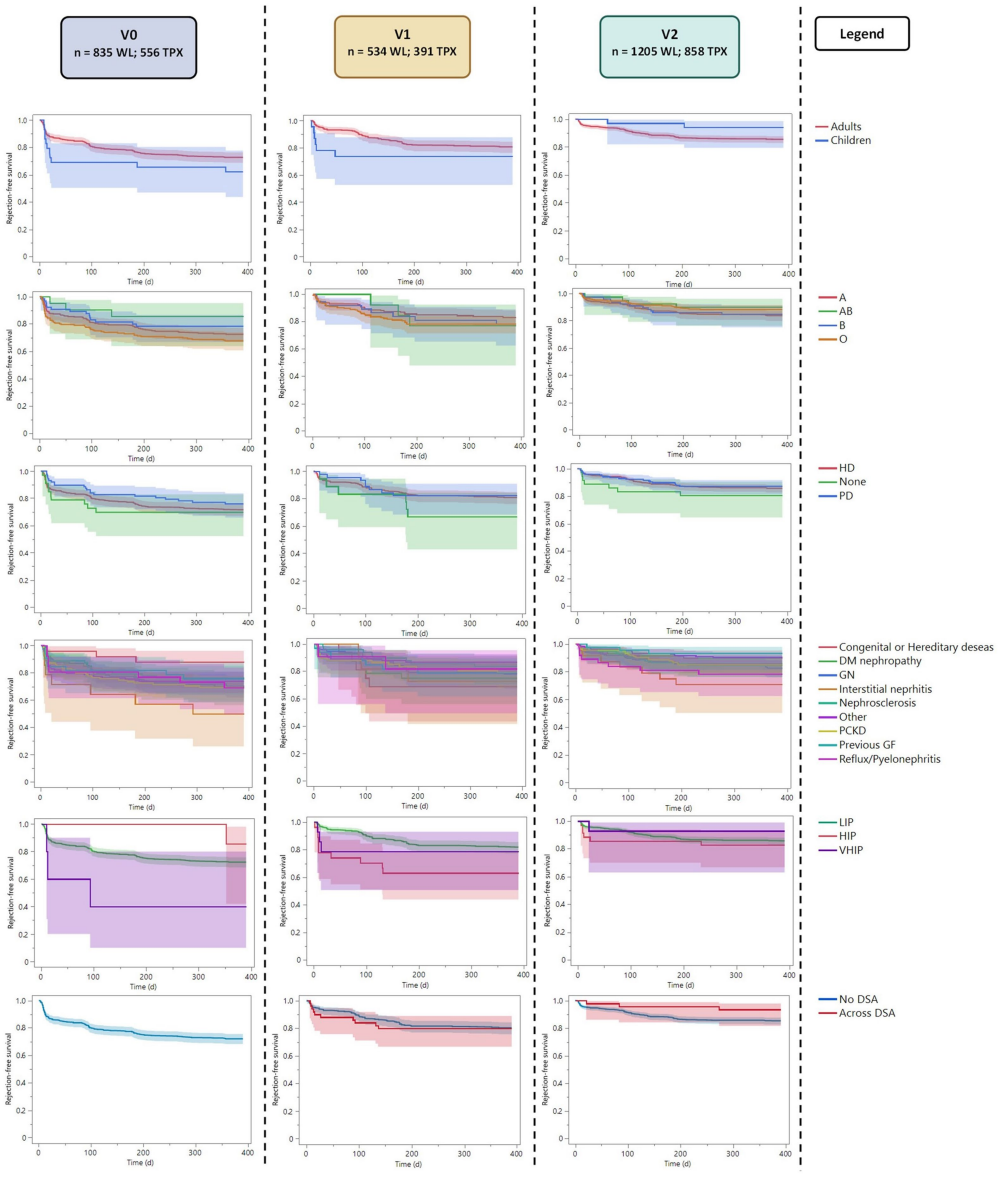
Kaplan–Meier plot with the time to rejection analysis (censured for first post-transplant-year rejection). LIR, Low Immunized Recipients (PRA <85); HIR, High Immunized Recipients (PRA ≤85 and < 98); VHIR, Very High Immunized Recipients (PRA ≥98).

The time-to-first rejection analysis (Table 2B; Figure 3) showed similar results in adults and children in the three periods (*p* = 0.172, p = 0.285, *p* = 0.160); similarly, blood group, dialysis modality, and diagnosis leading to ESKD presented with similar time-to-first rejection in the three periods (Supplementary Table S6). Considering the recipients’ immunization status, we observed a possible trend for faster rejection of highly immunized patients in v1 compared to non-immunized patients (*p* = 0.019). However, this was not statistically significant after the Bonferroni correction (*p* = 0.342). The time to rejection was similar between recipients with different immunization statuses in v0 and v2 (*p* = 0.107 and *p* = 0.648, respectively).

In v1, we observed more recipients transplanted across DSA (14%) than in v2 (6%). The mean cumulative MFI of recipients transplanted across DSA was similar in the two periods (4,518 ± 4,152 in v1 and 4,921 ± 3,477 in v2; *p* = 0.549). However, the presence of DSA at transplantation did not correlate with the time to first rejection in the two periods (Table 2B).

Considering the 1-year graft survival, only 74 recipients of the entire cohort presented with an early graft loss in the first year after transplantation, leading to a 1-year cohort graft survival of 97% (Table 1D). This value was similar in the three periods (Pearson’s chi-squared *p* = 0.053). In the three periods, no subgroup showed any significant difference in 1-year graft survival (Table 2C).

## Discussion

The Swiss allocation system for kidney transplantation has evolved from a more simplistic, waiting-time-based model with a better access to hyperimmunized recipients (based on the detection of anti-HLA antibodies and CDC cross-match) to a sophisticated, multi-faceted approach. This evolution has led to improvements driven by the necessity to increase the fairness of the allocation process for every category of patients (immunized, hyperimmunized, and non-immunized). Regular evaluation is necessary to demonstrate the importance of continually adapting transplantation policies to reflect medical advancements. As expected, our data showed that donors and recipients transplanted between 2007 and 2020 presented different characteristics, which makes a direct period comparison difficult. Many significant changes occurred in 2012 with the introduction of Luminex^®^, the use of a virtual crossmatch, and the progressive increase of CDC donors (introduced in 2011).

We noticed an increase in female recipients in v1 and v2, indicating either an increase in the number of females on the waiting list or better female access to transplantation. Our data did not permit us to distinguish between populational fluctuations over time or access on the list and to transplantation. The differences observed in blood groups and dialysis modality are probably associated with the fluctuations of characteristics of patients on the waiting list over time. A dedicated study assessing factors such as sex, gender, blood group, and dialysis modality, potentially limiting access to the waiting list, is still needed.

We observed faster transplantations in children than in adults in all three periods. This was expected, as the priority for children did not change in the allocation rules over the study period. Considering the access to transplantation and recipients’ ABO blood group, the system was not fair, showing significantly faster transplantation in group A recipients and slower transplantations for recipients of group 0 in the three periods. The impact of other blood groups was variable along the three periods, probably because of the variable number of compatible donors. In fact, the revisions of the allocation system did not consider modifications concerning the blood group, maintaining an iso-ABO allocation system. Considering these results, a change to an ABO-compatible system that includes the recipient blood group in the priority score could improve the fairness of organ allocations with potentially faster transplantation for non-A recipients.

Preemptive listing before 2012 (v0) resulted in faster transplantations, as allocation points were calculated from the moment of listing. After revising the allocation system in 2012, points were calculated from dialysis initiation, correcting this disparity v2 (and only partially in v1), even though reduced points could still be collected up to 18 months before the start of dialysis. The dialysis modality itself did not influence the time to transplantation.

Our data confirmed that introducing the cPRA and the priority score permitted the fair prioritization of hyperimmunized recipients when a matching organ was available (even in highly immunized patients). Since this revision, the allocation between low- and hyperimmunized recipients has remained balanced. This was one of the primary goals of the allocation system introduced in 2012. No correlation was observed between the time to transplantation in the three periods and the analysis of the cPRA as a continuous value. This is probably explained by the fact that only a minor number of cases with very high cPRA presented with significantly longer waiting times. The cause of ESKD and the presence of preformed DSA did not influence the time to transplantation.

The death rate on list decreased along the three periods, possibly because of the increase of available donors in relation to the increasing number of DCD donors. No difference was observed in the time-to-first rejection and 1-year graft survival in recipients of different blood groups, dialysis modality, or with preformed DSA. The comparison between low-immunized and hyperimmunized recipients (with both highly- and very highly-immunized recipients) showed no significant difference. These results are encouraging as no subgroups presented a higher risk for early graft loss with the current allocation system.

Finally, we could have expected longer rejection-free time after 2012 because of the introduction of the virtual crossmatch and the reduced number of transplants across DSA. However, the system considers only loci -A, -B, and -DR, giving each center the liberty to accept DSA against loci -C, -DQ, and -DP, and a recent publication of the STCS has demonstrated that a vast majority of the transplantations across DSA concern these loci (22).

The next revision of the allocation system of 2024 will include all loci except for DRB3/4/5. This could explain the lack of improvement in the time to rejection and one-year graft survival with the new algorithm.

## Conclusion

Our study showed that changes in the Swiss allocation system improved the fairness of access to transplantation for patients on the waiting list, particularly for hyperimmunized patients. On the other hand, our system still fails to grant fair access to recipients with different blood groups. For this reason, moving from an iso-blood group system to a compatible blood group system that includes the blood group in the priority score might improve the allocation fairness. Besides, our data showed that modifications of the allocation system did not negatively impact early post-transplant complications, maintaining a similar time to first rejection and 1-year graft survival in all analyzed subgroups. Such an analysis is helpful for retrospectively validating changes made to the allocation system and identifying aspects that need discussion for future revisions. A comprehensive analysis considering factors potentially limiting access to the waiting list is still needed.

## Limitations

The limited availability of donor’s clinical data prevented us from performing a competitive risk analysis between transplanted and non-transplanted patients, limiting the time to event analysis to patients who receive a transplantation. This comparison permitted the evaluation of the fairness between transplanted patients but might have excluded patients with a lower probability of being transplanted. The different characteristics and maximal theoretical waiting times of donors and patients on the waiting list and the different periods’ duration prevented the direct comparison of the mean waiting time across periods.

Finally, our study did not evaluate factors potentially limiting the access of patients to the waiting list and, consequently, to transplantation. We encourage a dedicated study in this direction.

## Data Availability

The data analyzed in this study is subject to the following licenses/restrictions: because of the STCS and SOAS data protection contract, data are not directly available but can be requested from the STCS Scientific Committee (FUP 163). Requests to access these datasets should be directed to https://www.stcs.ch/about/data-center.

## Footnotes

^1^ www.swisstransplant.org

^2^ https://www.swisstransplant.org/de/swisstransplant/jahresbericht

## References

[1] Tonelli M Wiebe N Knoll G Bello A Browne S Jadhav D. Systematic review: kidney transplantation compared with Dialysis in clinically relevant outcomes. Am J Transplant. (2011) 11:2093–109. doi: 10.1111/j.1600-6143.2011.03686.x, PMID: 21883901

[2] Abecassis M Bartlett ST Collins AJ Davis CL Delmonico FL Friedewald JJ. Kidney transplantation as primary therapy for end-stage renal disease: a National Kidney Foundation/kidney disease outcomes quality initiative (NKF/KDOQI™) conference. Clin J Am Soc Nephrol. (2008) 3:471–80. doi: 10.2215/CJN.05021107, PMID: 18256371 PMC2390948

[3] McCormick F Held PJ Chertow GM. The terrible toll of the kidney shortage. J Am Soc Nephrol. (2018) 29:2775–6. doi: 10.1681/ASN.2018101030, PMID: 30420419 PMC6287861

[4] Heemann U Oberbauer R Sprangers B Gökalp C Bemelman F. Deceased donor kidney allocation schemes and international exchange. Curr Opin Organ Transplant. (2020) 25:66–73. doi: 10.1097/MOT.0000000000000720, PMID: 31815789

[5] Wu DA Watson CJ Bradley JA Johnson RJ Forsythe JL Oniscu GC. Global trends and challenges in deceased donor kidney allocation. Kidney Int. (2017) 91:1287–99. doi: 10.1016/j.kint.2016.09.054, PMID: 28320531

[6] Cron DC Husain SA Adler JT. The new distance-based kidney allocation system: implications for patients, transplant centers, and organ procurement organizations. Curr Transplant Rep. (2022) 9:302–7. doi: 10.1007/s40472-022-00384-z, PMID: 36254174 PMC9558035

[7] Stegall MD Stock PG Andreoni K Friedewald JJ Leichtman AB. Why do we have the kidney allocation system we have today? A history of the 2014 kidney allocation system. Hum Immunol. (2017) 78:4–8. doi: 10.1016/j.humimm.2016.08.008, PMID: 27554430

[8] Mayer G Persijn GG. Eurotransplant kidney allocation system (ETKAS): rationale and implementation. Nephrol Dial Transpl. (2006) 21:2–3. doi: 10.1093/ndt/gfi269, PMID: 16287913

[9] Audry B Savoye E Pastural M Bayer F Legeai C Macher MA. The new French kidney allocation system for donations after brain death: rationale, implementation, and evaluation. Am J Transplant. (2022) 22:2855–68. doi: 10.1111/ajt.17180, PMID: 36000787

[10] Sethi S Najjar R Peng A Mirocha J Vo A Bunnapradist S. Allocation of the highest quality kidneys and transplant outcomes under the new kidney allocation system. Am J Kidney Dis. (2019) 73:605–14. doi: 10.1053/j.ajkd.2018.12.036, PMID: 30929853

[11] Lieber SR Perez FVT Tabossi MR Persoli LBL Marques SBD Mazzali M. Effect of panel-reactive antibody in predicting crossmatch selection of cadaveric kidney recipients 39:429–31. doi: 10.1016/j.transproceed.2007.01.045, PMID: 17362748

[12] Terasaki PI McClelland JD. Antibody response to homografts: VIII. Relation of mouse hemagglutinins and cytotoxins. J Exp Med. (1963) 117:675–90. doi: 10.1084/jem.117.4.675, PMID: 13980634 PMC2137615

[13] Tait BD Hudson F Cantwell L Brewin G Holdsworth R Bennett G. Review article: Luminex technology for HLA antibody detection in organ transplantation 14:247–54. doi: 10.1111/j.1440-1797.2008.01074.x 19207861

[14] Cecka JM Calculated PRA. (CPRA): the new measure of sensitization for transplant candidates: special feature. Am J Transplant. (2010) 10:26–9. doi: 10.1111/j.1600-6143.2009.02927.x, PMID: 19958328

[15] Lefaucheur C Loupy A Hill GS Andrade J Nochy D Antoine C. Preexisting donor-specific HLA antibodies predict outcome in kidney transplantation 21:1398–407. doi: 10.1681/ASN.2009101065 PMC2938596 20634297

[16] Riethmüller S Ferrari-Lacraz S Müller MK Raptis DA Hadaya K Rüsi B. Donor-specific antibody levels and three generations of crossmatches to predict antibody-mediated rejection in kidney transplantation. Transplantation. (2010) 90:160–7. doi: 10.1097/TP.0b013e3181e36e08, PMID: 20658760

[17] Weiss J Beyeler F Immer FF. Heart allocation and transplantation in Switzerland since the introduction of the Swiss organ allocation system (SOAS). Swiss Med Wkly. (2014) 144:w14057–9. doi: 10.4414/smw.2014.14057, PMID: 27922169

[18] Klinzing S Brandi G Raptis DA Wenger U Weber D Stehberger PA. Influence on ICU course, outcome and costs for lung transplantation after implementation of the new Swiss transplantation law 3:1–8. doi: 10.1186/2047-1440-3-9 PMC3975267 24690254

[19] Oberkofler CE Stocker R Raptis DA Stover JF Schuepbach RA Müllhaupt B. Same quality - higher price? The paradox of allocation: the first national single center analysis after the implementation of the new Swiss transplantation law: the ICU view. Clin Transpl. (2011) 25:921–8. doi: 10.1111/j.1399-0012.2010.01364.x, PMID: 21108659

[20] Koller MT Van Delden C Müller NJ Baumann P Lovis C Marti H-P. Design and methodology of the Swiss transplant cohort study (STCS): a comprehensive prospective nationwide long-term follow-up cohort. Eur J Epidemiol. (2013) 28:347–55. doi: 10.1007/s10654-012-9754-y, PMID: 23546766 PMC3653038

[21] Heidt S Haasnoot GW van der Linden-van Oevelen MJH Claas FHJ. Highly sensitized patients are well served by receiving a compatible organ offer based on acceptable mismatches. Front Immunol. (2021) 12:12. doi: 10.3389/fimmu.2021.687254, PMID: 34248971 PMC8267476

[22] Frischknecht L Deng Y Wehmeier C de Rougemont O Villard J Ferrari-Lacraz S. The impact of pre-transplant donor specific antibodies on the outcome of kidney transplantation – data from the Swiss transplant cohort study. Front Immunol. (2022) 13:13. doi: 10.3389/fimmu.2022.1005790, PMID: 38361942 PMC10867099

