# Supplementary material for: Assessment of access and outcomes of kidney transplantation through the reforms of the Swiss organ allocation system
Source: Front Public Health. 2025 Jan 8;12:1500781. doi: 10.3389/fpubh.2024.1500781 (PMC11751015; doi:10.3389/fpubh.2024.1500781)
Supplement: Supplementary file 1 [file Supplementary_file_1.docx]

Supplementary Materials

- **Supplementary Table** **1**: Summary of the allocation rules changes throughout the three periods …...P2
- **Supplementary Table 2:** Priority Score ………………………………………………………………P3
- **Supplementary Table 3:** List of variables available from the STCS and SOAS databases......……....P4
- **Supplementary Table 4**: Bonferroni correction of the time-to-event analysis …………………….....P5
- **Supplementary Table 5:** Time-to-transplantation hazard ratio ……………………………...……….P6
- **Supplementary Table 5:** Time-to-rejection hazard ratio …………………….……………...………..P7
- **Supplementary Figure 1**: Simulations and validation of the cPRA score …..........................……….P8

| **Period** | **Year** | **Changes in allocation rules** |
| --- | --- | --- |
| v0 | 01.07.**2007** - 30.06.**2012** | Initial rules without virtual crossmatch |
| v1 | 01.07.**2012** - 31.05.**2015** | Introduction of CDC Donors in the allocation system  Introduction of Luminex® and the use of a virtual crossmatch with a calculated PRA (cPRA).  Introduction of the *Priority Score* (defined in Supplementary Table 3).  Introduction of the concept of accepted DSA for highly immunized patients. |
| v3 | 01.06.**2015** - 24.11.**2017** | Adjustment of the cPRA Score. The constant *a,* multiplying the cPRA, was reduced from 120 to 84 to reduce the gap between the offer and demand of patients with and without hyperimmunization (based on the estimation model shown in Supplementary Figure 1). |
|  | 25.11.**2017** - 31.12.**2019** | Adjustment of the waiting time score with limited maximal points allocated to patients with preemptive listing (max 18 months). |

**Supplementary Table 1**: Summary of the allocation rules changes throughout the three study periods

**Priority Score**

| **Waiting Time Score + HLA Match Score + cPRA Score** |
| --- |
| Waiting Time Score = (c*Mt on list before HD + d*Mt on List since HD) /12  HLA Match Score = 0.333*A Match + 0.333*B Match + 1.000 * DR Match  cPRA Score = a*cPRA + (100-cPRA)*b |

***Supplementary Table 2:*** *Model of the calculation of the priority score. The variables have been adapted along with the different law revisions. After the 2015 revision, 1x HLA-A or B match was equivalent to 4 months waiting time, and 1x HLA-DR match was equivalent to 1 year waiting time. A cPRA of 100% was equivalent to 3 years waiting time.*

**List of variables available from the STCS and SOAS databases**

| **Data from Swiss Transplantation Cohort Study (STCS)** | **Data from the Swiss Organ Allocation System**  **(SOAS)** |
| --- | --- |
| **Recipient** | **Patients on the waiting list** |
|  |  |
| - Age | - Date of list removal |
| - Sex | - Reason for list removal (transplant vs. other reason) |
| - Blood group | - Recipient HLA typing |
| - Reference center | - Recipient DSA and MFI |
| - Transplant date | - Pick PRA |
| - Listing date |  |
| - Dialysis modality |  |
| - Basic pathology |  |
| - HLA typing |  |
| - Transplant complications |  |
| - Kind of complications |  |
| - Complication date |  |
| - Graft loss date |  |
| - Rejection date |  |
| - Biopsy date |  |
| - Biopsy results |  |
|  |  |
|  |  |
| **Donor** | **Donor** |
|  |  |
| - Donor type | - Blood group |
|  | - Age |
|  | - Cause of death |
|  | - HLA Typing |

**Supplementary Table 3**: List of variables available through the two databases, the Swiss Transplantation Cohort Study and the Swiss Allocation System.

|  |  |  | | |
| --- | --- | --- | --- | --- |
|  | | **Historical periods**  **(corrected p-values)** | | |
|  | | **v0** | **v1** | **v2** |
| **(A) Time to transplant** | |  |  |  |
|  | Age | **<0.01** | **<0.01** | **<0.01** |
|  | ABO blood group | **<0.01** | **<0.01** | **<0.01** |
|  | ESKD diagnosis | 1 | 1 | 0.522 |
|  | Dialysis modality | **<0.01** | **0.005** | 0.846 |
|  | Immunity group | **<0.01** | 1 | 0.252 |
|  | Transplantation across DSA | - | 1 | 1 |
|  |  |  |  |  |
| **(B) Time to Rejection** | |  |  |  |
|  | Age | 1 | 1 | 1 |
|  | ABO blood groups | 1 | 1 | 1 |
|  | ESKD diagnosis | 1 | 1 | 1 |
|  | Dialysis modality | 1 | 1 | 1 |
|  | Immunity group | 1 | 0.342 | 1 |
|  | Transplantation across DSA | - | 1 | 1 |
|  |  |  |  |  |
| **D) 1-Y graft survival** |  |  |  |  |
|  | Age | 1 | 1 | 1 |
|  | ABO blood group | 1 | 1 | 1 |
|  | ESKD diagnosis | 1 | 1 | 1 |
|  | Dialysis modality | 1 | 1 | 1 |
|  | Immunity group | 1 | 1 | 1 |
|  | Transplantation across DSA | - | 1 | 1 |
|  |  |  |  |  |

**Supplementary Table 4:** Bonferroni correction showing the corrected p-values of the time-to-event analysis considering (A) time to transplantation (Log-Rank test), (B) time to rejection (Log-Rank test), and (C) 1Y-graft survival analysis (Pearson’s Chi-Square test). A p-value of 0.05 was considered as significant.

|  | **V0** | | **V1** | | **V3** | |
| --- | --- | --- | --- | --- | --- | --- |
| **Level** | **Hazard-Ratio** | **ChiSq** | **Hazard-Ratio** | **ChiSq** | **Hazard-Ratio** | **ChiSq** |
| **Age Group** |  |  |  |  |  |  |
| Child - Adult | 7.44 (4.95-11.19) | **<.0001** | 15.89 (9.17-27.5) | **<.0001** | 7.42 (5.08-10.81) | **<.0001** |
|  |  |  |  |  |  |  |
| **ABO Group** |  |  |  |  |  |  |
| A-AB | 9.96 (0.60-1.51) | 0.844 | 1.70 (0.95-3.04) | 0.0764 | 2.20 (1.59-3.05) | **<.0001** |
| A-B | 2.33 (1.75-3.12) | **<.0001** | 1.82 (1.23-2.69) | **0.003** | 2.39 (1.88-3.05) | **<.0001** |
| A-O | 4.52 (3.61-5.65) | **<.0001** | 3.92 (3.00-5.08) | **<.0001** | 2.24 (1.91-2.62) | **<.0001** |
| AB-B | 2.44 (1.47-4.07) | **<.001** | 1.07 (0.55-2.08) | 0.833 | 1.09 (0.75-1.59) | 0.659 |
| AB-O | 4.73 (2.92-7.69) | **<.0001** | 2.31 (1.29-4.11) | **0.005** | 1.02 (0.73-1.41) | 0.918 |
| B-O | 1.94 (1.44-2.60) | **<.0001** | 2.15 (1.44-3.20) | **<.001** | 0.94 (0.73-1.20) | 0.593 |
|  |  |  |  |  |  |  |
| **Dialysis modality** |  |  |  |  |  |  |
| HD-PD | 1.54 (1.21-1.97) | **<.001** | 1.00 (.0.72-1.37) | 0.976 | 1.15 (0.96-1.37) | 0.123 |
| None-HD | 1.73 (1.20-2.48) | **0.003** | 1.30 (0.76-2.21) | 0.340 | 0.86 (0.62-1.20) | 0.386 |
| None-PD | 2.66 (1.75-4.05) | **<.0001** | 1.29 (0.72-2.32) | 0.396 | 0.99 (0.70-1.41) | 0.971 |
|  |  |  |  |  |  |  |
| **Immunity Group** |  |  |  |  |  |  |
| Low-Immunized  Highly immunized | 3.95 (1.81-8.59) | **<.001** | 0.81 (0.51-1.30) | 0.381 | 1.24 (0.89-1.74) | 0.205 |
| Low-immunized  Very highly immunized | 10.40 (3.28-33.0) | **<.0001** | 1.63 (0.86-3.09) | 0.136 | 1.39 (0.82-2.37) | 0.713 |
| Highly immunized  Very highly immunized | 2.64 (0.67-10.42) | 0.167 | 2.01 (1.02-3.97) | **0.044** | 1.12 (0.62-2.03) | 0.713 |
|  |  |  |  |  |  |  |
| **Transplant Across DSA** |  |  |  |  |  |  |
| Across DSA  No DSA | - | - | 1.00 (0.71-1.41) | 0.990 | 0.94 (0.69-1.30) | 0.718 |
|  |  |  |  |  |  |  |

**Supplementary Table 5:** Active time-to-transplantation hazard ratio (with 95% confidence interval ) and ChiSq for active time-to-transplantation for each subgroup along the three periods.

|  | **V0** | | **V1** | | **V3** | |
| --- | --- | --- | --- | --- | --- | --- |
| **Level** | **Hazard-Ratio** | **ChiSq** | **Hazard-Ratio** | **ChiSq** | **Hazard-Ratio** | **ChiSq** |
| **Age Group** |  |  |  |  |  |  |
| Child - Adult | 1.65 (0.83-3.28) | 0.153 | 1.61 (0.63-4.10) | 0.318 | 0.44 (0.10-1.96) | 0.283 |
|  |  |  |  |  |  |  |
| **ABO Group** |  |  |  |  |  |  |
| A-AB | 2.27 (0.71-7.28) | 0.169 | 0.56 (0.16-1.90) | 0.350 | 1.65 (0.60-4.57) | 0.334 |
| A-B | 1.40 (0.79-2.49) | 0.251 | 0.56 (0.16-1.90) | 0.360 | 1.10 (0.59-2.04) | 0.776 |
| A-O | 0.82 (0.58-1.15) | 0.248 | 0.72 (0.43.1.22) | 0.220 | 1.41 (0.93-2.14) | 0.109 |
| AB-B | 0.62 (0.18-2.17) | 0.452 | 1.23 (0.32-4.79) | 0.765 | 0.66 (0.21-2.07) | 0.480 |
| AB-O | 0.36 (0.11-1.17) | 0.089 | 1.30 (0.39-4.33) | 0.675 | 0.85 (0.30-2.41) | 0.763 |
| B-O | 0.58 (0.32-1.05) | 0.073 | 1.05 (0.47-2.34) | 0.900 | 1.29 (0.66-2.50) | 0.459 |
|  |  |  |  |  |  |  |
| **Dialysis Type** |  |  |  |  |  |  |
| HD-PD | 1.39 (0.86-2.24) | 0.184 | 1.04 (0.49-2.26) | 0.906 | 1.14 (0.70-1.87) | 0.598 |
| None-HD | 1.22 (0.62-2.43) | 0.564 | 2.26 (0.92-5.55) | 0.075 | 1.70 (0.77-3.75) | 0.191 |
| None-PD | 1.70 (0.77-3.74) | 0.191 | 2.37 (0.80-6.98) | 0.118 | 1.94 (0.81-4.65) | 0.138 |
|  |  |  |  |  |  |  |
| **Immunity Group** |  |  |  |  |  |  |
| LIP-HIP | 2.44 (00.34-17.61) | 0.375 | 0.34 (0.17-0.71) | **0.004** | 0.71 (0.30-1.67) | 0.430 |
| LIH-VHIP | 0.36 (0.11-1.21) | 0.100 | 0.76 (0.20-2.88) | 0.681 | 1.35 (0.18-9.92) | 0.771 |
| HIP-VHIP | 0.15 (0.02-1.50) | 0.106 | 2.21 (0.53-9.14) | 0.275 | 1.90 (0.23-15.97) | 0.555 |
|  |  |  |  |  |  |  |
| **Across DSA** |  |  |  |  |  |  |
| DSA –  No DSA | - | - | 1.04 (0.48-2.23) | 0.926 | 0.42 (0.13-1.38) | 0.151 |
|  |  |  |  |  |  |  |

**Supplementary Table 6:** Time-to-rejection hazard ratio (with 95% confidence interval) and ChiSq for time-to-transplantation for each subgroup along the three periods. Censured for rejection in the first year post-transplantation.

***
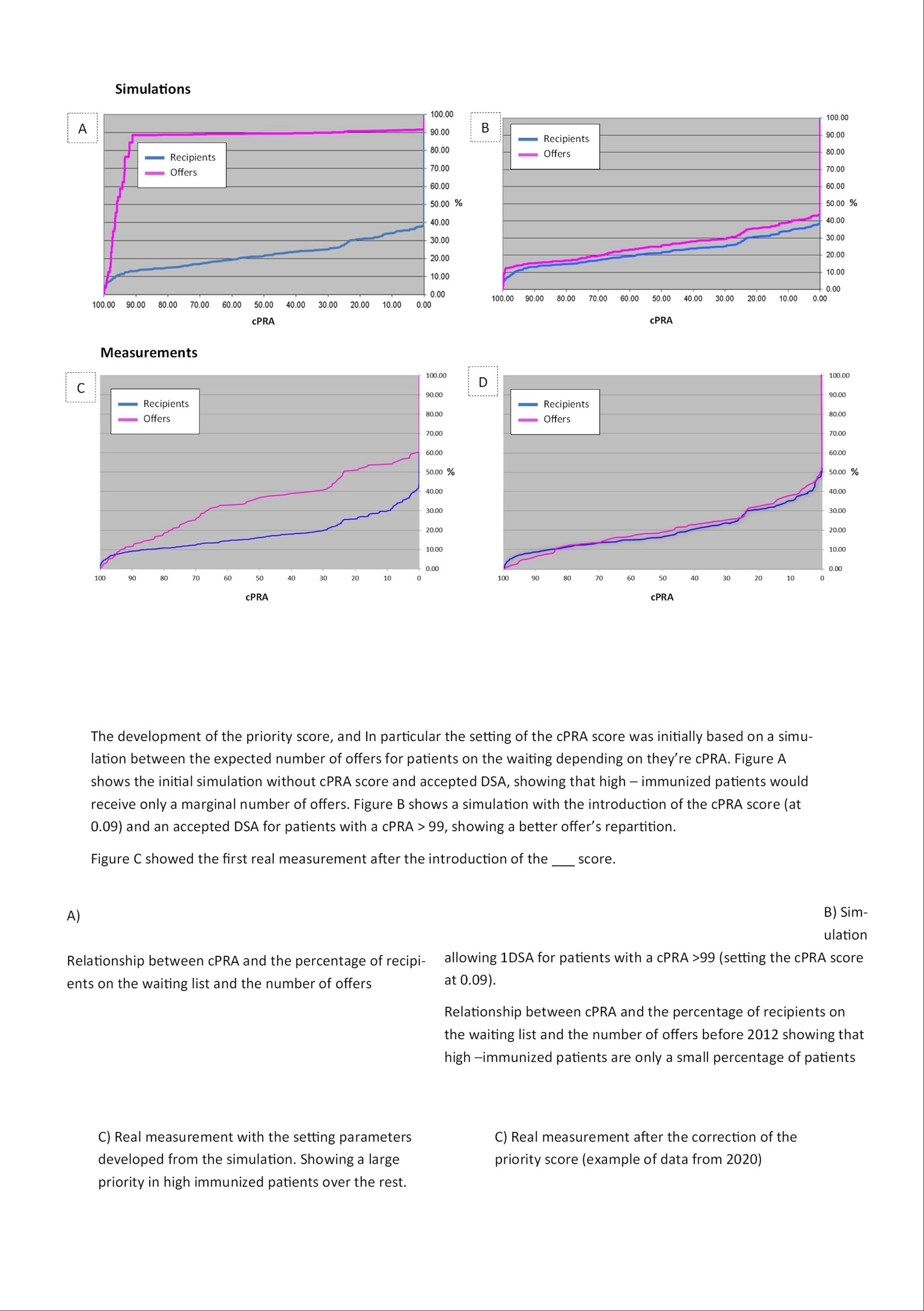
***

**Supplementary Figure 1:** The development of the priority score and the calibration of the cPRA score were initially based on a simulation between the expected number of offers for patients on the waiting list depending on their cPRA. Figure A shows the initial simulation without a cPRA score and accepted DSA, showing that hyperimmunized patients would receive only a marginal number of offers. Figure B shows a simulation after introducing the cPRA score (at 0.09) and 1 accepted DSA for patients with a cPRA > 99%, showing a better repartition of offers. Figure C shows the real measurement after introducing the priority score, possibly over prioritizing hyperimmunized patients. Figure D shows the repartition of offers over recipients’ cPRA after correcting the priority score in 2015.
